# The Pharmacological Activity of Non-Cannabinoid Phytochemicals in *Cannabis sativa* L.: A Systematic Review

**DOI:** 10.3390/plants15142142

**Published:** 2026-07-11

**Authors:** Olakunle Sanni, Modupe Olufunmilayo Ogunrombi, Chikwelu Lawrence Obi

**Affiliations:** 1 Department of Clinical Pharmacology and Therapeutics, School of Medicine, Sefako Makgatho Health Sciences University, Pretoria 0204, South Africa; 2 Department of Biology and Environmental Sciences, School of Science and Technology, Sefako Makgatho Health Sciences University, Pretoria 0204, South Africa; lawrence.obi@smu.ac.za

**Keywords:** *Cannabis sativa*, non-cannabinoid, phytochemical, pharmacological activity, medicinal plants

## Abstract

*Cannabis sativa* (*C. sativa*) is widely recognized for its therapeutic potential, historically attributed to its cannabinoid content. Some of the phytocannabinoid compounds of *C. sativa* are mediated through interactions with the endocannabinoid system, while some are able to modulate the interaction between cannabinoids and the endocannabinoid system. Additionally, the non-cannabinoid group exhibits a broad range of bioactive potential, also displaying pharmacological actions. However, the non-cannabinoid fraction of the plant has not been extensively studied. This review emphasizes the emerging pharmacological importance of non-cannabinoid phytochemicals such as terpenes, flavonoids, phenolic compounds, and alkaloids found within *C. sativa*. Scopus, Google Scholar, and PubMed were utilized as databases to search for relevant published literature. The search employed the keywords such as “*Cannabis sativa* phytochemicals, non-cannabinoid compounds of *Cannabis sativa*, pharmacological activity of non-cannabinoid compounds of *Cannabis sativa*”. The total number of published articles initially retrieved between January 2010 and May 2025 was 187. From these, only 12 articles were selected according to the inclusion and exclusion criteria. The review highlights the structural diversity and functional significance of non-cannabinoid constituents. Terpenes and flavonoids were particularly well-characterized, with demonstrated synergistic interactions that enhanced therapeutic efficacy via mechanisms like the “entourage effect.” Despite promising preclinical findings, the clinical translation of these compounds remains limited due to challenges in standardization, regulatory barriers, and a lack of well-defined analytical methods. This review concludes that non-cannabinoid phytochemicals are integral to the pharmacological complexity of *C. sativa* and warrant further investigation as potential candidates for drug development, especially in formulations that aim to optimize whole-plant therapeutic effects.

## 1. Introduction

*Cannabis sativa* belongs to the *Cannabaceae* family and has attracted a lot of interest lately because of its psychoactive effects [1]. Despite the divergent opinions on the use of *C. sativa*, it has been widely employed across various cultures and regions to address numerous health challenges, such as arthritis, diabetes, pain, liver disease, cancer, inflammation, cardiovascular diseases, metabolic syndrome, and gastrointestinal disorders [2,3].

Recently, *C. sativa* has been used as a nutraceutical and cosmetic agent. For instance, oil from seeds is widely used due to their nutritional value and skincare benefits [4]. Also, the seed has been utilized in agriculture for animal bedding and as a source of animal feed [5]. These medicinal and nutraceutical benefits of *C. sativa* are largely linked to its cannabinoids and other bioactive compounds.

Over 500 phytochemicals containing arrays of different classes of biochemicals, such as phenolics, cannabinoids, terpenes, and flavonoids, to mention a few, have been identified in *C. sativa* [6]. Cannabinoids have attracted more attention due to their association with cannabis and their interaction with cannabinoid and non-cannabinoid receptors, as well as other pharmacological targets. In addition, the psychotic effects of Δ9-tetrahydrocannabinol, a cannabinoid found in *C. sativa*, have been studied in more detail compared to the non-cannabinoid compounds.

The non-cannabinoid phytochemicals are made up of terpenes, flavonoids, glycosides, alcohols, steroids, simple acids, and phenols [7] and they have been reported to possess an array of pharmacological and biological activities in many plants, including *C. sativa*. Some of the activities include antioxidant, anti-inflammatory, analgesic, antimicrobial, anti-diabetic, and many others [8,9].

However, there is a paucity of information on the pharmacological and biological activity of non-cannabinoid phytochemicals belonging to *C. Sativa*. Most information centered on the cannabinoid component of the plant, thus undermining the therapeutic potential of the plant’s non-cannabinoid phytochemicals. Therefore, this review aims to explicate the non-cannabinoid phytochemicals found in *C. sativa* with emphasis on their pharmacological activity.

## 2. Ethnobotanical Uses of *Cannabis sativa*

**Figure 1:**
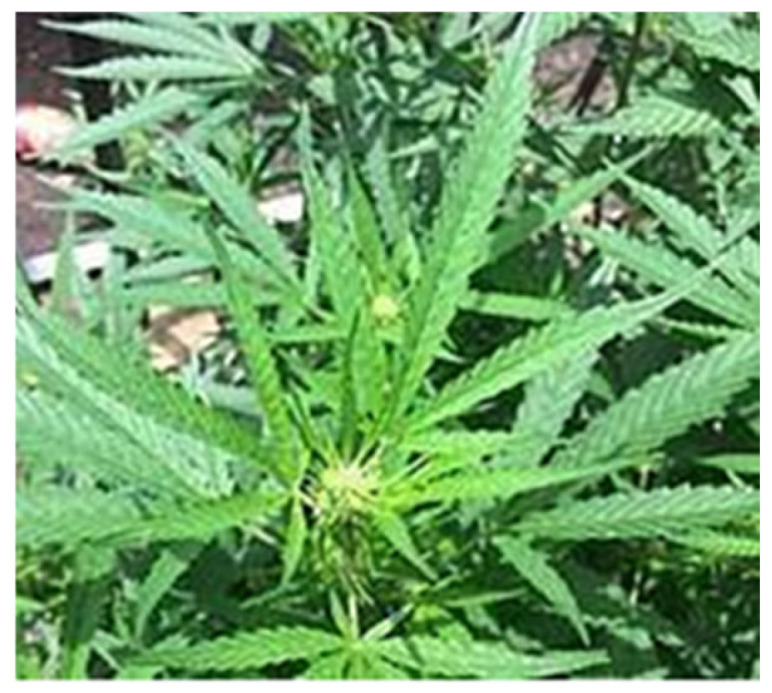
*Cannabis sativa* plant [15].

*Cannabis sativa*, commonly referred to as hemp or marijuana (Figure 1), has been integral to human societies since early domestication, with data confirming its use for more than ten millennia [10,11]. Its ethnobotanical value is reflected in its diverse roles in traditional medicine, nutrition, cultural practices, and folklore. The folklore usage of *C. sativa* has been associated with mystical and spiritual practices. For example, *C. sativa* leaves are reportedly used in ritualistic practices during festivals such as Holi and Shivaratri in India [12], and for spiritual ecstasy and divine connection in the Middle East and North Africa [13]. In the Sahara, Africa, cannabis leaves were believed to ward off evil spirits, while leaf infusions were used in divination and traditional healing ceremonies [14].

The ethnopharmacological evidence, dating back to 1800 BCE, highlights *C. sativa* as an analgesic, sedative, anti-inflammatory, and antispasmodic agent [16,17]. Decoctions and infusions from leaves, seeds, and flowers were traditionally administered to treat epilepsy, insomnia, malaria, and rheumatism [11,18]. Historically, *C. sativa* was made into flour, roasted, or pressed for oil and consumed as food due to its protein-rich content [19,20].

In addition, *C. sativa* is used for producing ropes, sails, fishing nets, and textiles. Archeological findings from ancient China and Europe show hemp paper production dating back to the first centuries [21]. Traditional communities also utilized cannabis oil for lighting lamps, soap-making, and as a varnish [22].

## 3. Overview of *Cannabis sativa* Phytochemicals

The secondary metabolites in plants are a diverse group of phytochemicals that are not directly involved in the primary physiological processes of reproduction, growth, or development [23]. In contrast to primary metabolites such as amino acids, carbohydrates, and nucleotides, secondary metabolites perform specialized ecological functions, including defense against herbivores and pathogens [24], interspecies signaling [25], and adaptation to abiotic stress [26].

*C. sativa* synthesizes a wide spectrum of phytochemicals, both cannabinoids and non-cannabinoid compounds, classified as secondary metabolites that, while not required for primary physiological processes, play key roles in ecological interactions and exhibit considerable pharmacological relevance [23].

### 3.1. Cannabinoid Phytochemicals in Cannabis sativa

The cannabinoid compounds are a unique class of C_21_ terpenophenolic compounds predominantly synthesized in the glandular trichomes of flowers and leaves, but exert their biological target mainly on endocannabinoid receptors [27]. Examples of well-studied cannabinoids include Δ^9^-tetrahydrocannabinol (Δ^9^-THC), cannabigerol (CBG), cannabidiol (CBD), cannabinol (CBN), tetrahydrocannabivarin (THCV), and cannabichromene (CBC). Meanwhile, non-cannabinoid phytochemicals are distributed across different plant parts and interact with diverse molecular targets, including enzymes, ion channels, and non-cannabinoid receptors [28].

The therapeutic relevance of cannabinoid phytochemicals has been demonstrated in different disease models by a number of studies. For example, the principal psychoactive constituent of *C. sativa*, Δ^9^-THC, has been reported to be effective in pain management, antiemesis, appetite stimulation, and reducing spasticity in multiple sclerosis and spinal cord injury [29,30,31,32]. Non-psychoactive cannabinoid, CBD, is recognized for its antipsychotic, anti-inflammatory, antioxidant, and neuroprotective effects [33]. The multifaceted pharmacological effects of the cannabinoid compounds of *C. sativa* are mediated through interactions with the endocannabinoid system (ECS) [34], although it also interacts with non-cannabinoid targets.

### 3.2. Non-Cannabinoid Phytochemicals in Cannabis sativa

A diverse group of phytochemicals belonging to the non-cannabinoid group has been reported to include flavonoids, alkaloids, phenols, and terpenes [7]. These compounds add to the intricate array of secondary metabolites in *C. sativa* [6].

#### 3.2.1. Flavonoids

Flavonoids are a major class of polyphenolic phytochemicals that are widely distributed in the plant kingdom [23]. In *C. sativa*, approximately 20 flavonoids, predominantly within the flavonol and flavone subclasses, have been identified [35]. These compounds contribute to the plant’s pigmentation, UV protection, and defense, and are increasingly recognized for their therapeutic properties in humans. Flavonoid concentrations vary across plant tissues, with notable induction under abiotic stress (e.g., UV-C light), indicating the role in adaptive stress responses of flavonoids [23].

**Figure 2:**
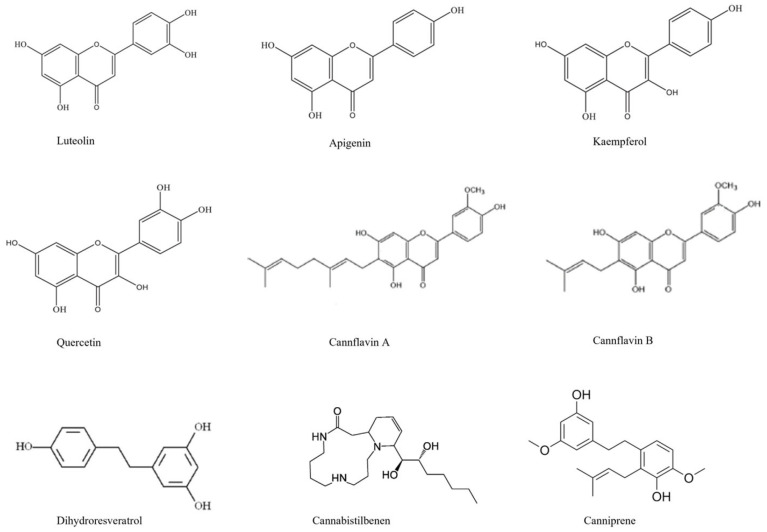
Flavonoids found in *C. sativa*.

Flavonoids are also low molecular weight polyphenolic compounds with a 15-carbon skeleton arranged in a C6-C3-C6 configuration [36]. They are classified into six main subclasses: flavonols, flavones, flavanones, isoflavones, flavanols (catechins), and anthocyanins, as shown in Figure 2. Different classes of flavonoids have been identified in *C. sativa.* Apigenin, luteolin, kaempferol, and quercetin, typically present in their *O*-glycosylated forms, have been identified as flavones and flavanols [37]. Cannflavin A and B are isoflavones exclusive to *C. sativa* [38]. Cannabistilbenes and dihydroresveratrol are dihystilbenoids found in the seeds and roots of cannabis [39]. They are primarily dihydrostilbenoids, and many are prenylated flavonoids. Recently, Canniprene B has been isolated from the leaves of *C. sativa* and identified as a prenylated dihydrostilbene [40].

#### 3.2.2. Alkaloids

**Figure 3:**
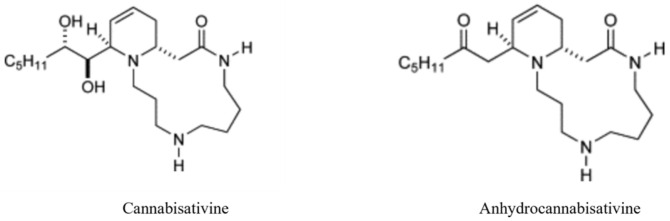
Alkaloids found in *C. sativa*.

Alkaloids constitute a diverse group of nitrogen-containing heterocyclic organic compounds, typically featuring one or more nitrogen atoms integrated within their ring structures. In addition to nitrogen, these molecules may incorporate other heteroatoms such as oxygen, sulfur, chlorine, bromine, or phosphorus. Although predominantly associated with plant metabolism, alkaloids are also biosynthesized by various microorganisms and animal species [41]. In the plant kingdom, they primarily function as chemical defense agents against herbivory [42]. A significant proportion of alkaloids exhibit potent pharmacological activity, accounting for approximately 60% of therapeutics derived from plant sources. Also, alkaloids are believed to act as modulators for growth and development in plants. The earliest alkaloid to be identified in the ethanol extract of *C. sativa* root (Figure 3) is spermidine-type alkaloids, namely, cannabisativine and anhydrocannabisativine [43]. However, their structures and pharmacological profiles are poorly characterized.

#### 3.2.3. Phenols

**Figure 4:**
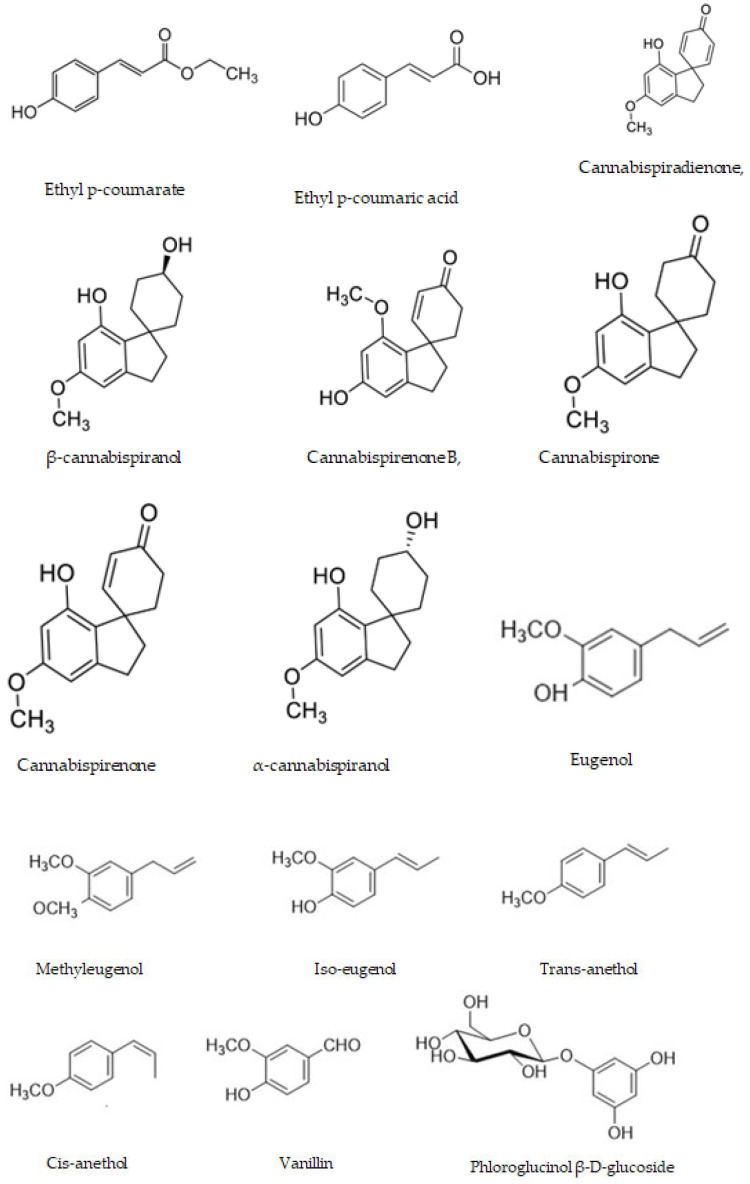
Phenols found in *C. sativa*.

Non-cannabinoid phenols encompass a wide range of chemical classes, including phenolic acids, spiro cannabinoid (spiroindan), and simple phenols as shown in Figure 4.

Phenolic acids are a class of non-flavonoid polyphenolic metabolites, mainly divided into two sub-groups: hydroxybenzoic and hydroxycinnamic acid, distinguished by their C1–C6 and C3–C6 structural frameworks, respectively [44]. They are water-soluble with a profound antioxidant activity, antitumor, anti-obesity, anti-cancer, and anti-diabetic properties [45,46]. The potential activity has been attributed to the phenyl rings with six-carbon aromatic structures arranged in a hexagonal configuration, where each of the five carbon atoms is bonded to a single hydrogen atom, contributing to the molecule’s resonance stability and electrophilic substitution reactivity. The phenolic acid profile of *C. sativa* includes chlorogenic acid, hydroxycinnamic acids, ferulic acid, and caffeic acid [47,48,49]. Recently, ethyl *p*-coumarate and *p*-coumaric acid have been identified as the main phenolic compounds found in aqueous and ethanol extracts of *C. sativa* roots [47].

Spiroindans are a class of non-cannabinoid phenols distinguished by a core structure comprising a benzene ring fused to a cyclopentyl group, which is further linked to a cyclohexane moiety in a spiro arrangement [35]. Six spiro cannabinoids have been isolated from the leaves of C. sativa as fraction B in a systematic extraction using water and acetone as eluting solvents on an HPLC-assisted purification column. These six compounds are cannabispiradienone, β-cannabispiranol, cannabispirenone B, cannabispirone, cannabispirenone, and α-cannabispiranol [50].

The non-cannabinoid simple phenols are reported to be present in the essential oil of Cannabis [51]. Six compounds of simple phenols have been isolated from the hemp pectin. They are eugenol, methyleugenol, iso-eugenol, trans-anethol, cis-anethol, and vanillin [52] while Phloroglucinol β-D-glucoside was isolated from the stem [53].

#### 3.2.4. Terpenes

Terpenes represent a bioactive and structurally diverse class of phytochemicals distributed widely in the plant kingdom [54]. In *C. sativa*, over 100 distinct terpenes have been identified, many of which are synthesized and accumulated within glandular trichomes, a specialized epidermal structure that also serves as the primary site for cannabinoid biosynthesis. Terpenes contribute significantly to the organoleptic properties of *C. sativa* and are increasingly recognized for their pharmacological activity and potential synergy with cannabinoids [54].

Terpenes are generally grouped on the basis of the number of isoprene units. Monoterpenes such as D-limonene, linalool, β-myrcene, and α-pinene are characterized by having two isoprene units, while triterpenes, primarily found in roots, fibers, and seeds, are characterized by having six isoprene units. Examples of triterpenes are β-amyrin and cycloartenol. Terpenes are produced in cannabis trichomes together with the cannabinoids, and are known for the plant’s distinct smell [55].

**Figure 5:**
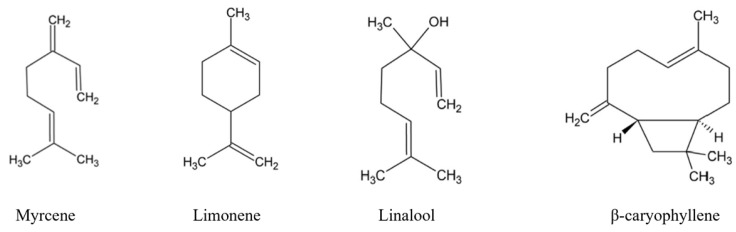
Terpenes found in *C. sativa*.

Terpenes are widely known for their synergistic interaction with various phytochemicals within *C. sativa*, especially the cannabinoids, to modulate and enhance the overall therapeutic efficacy. This is called “entourage effect,” as first introduced by Mechoulam and Ben-Shabat in 1998 [56]. This effect challenges the traditional single-compound drug model and supports the use of whole-plant extracts in phytotherapy. For example, terpenes such as myrcene, limonene, linalool, and β-caryophyllene, (Figure 5) have been shown to influence the pharmacokinetics, receptor affinity, and bioavailability of cannabinoids like Δ^9^-THC and cannabidiol (CBD) [57]. These interactions can result in enhanced efficacy, reduced adverse effects, and improved patient outcomes. Also, myrcene is believed to facilitate the crossing of cannabinoids across the blood–brain barrier, potentially enhancing the central effects of Δ^9^-THC [33].

#### 3.2.5. Fatty Acids

Fatty acids possess significant nutritional importance and constitute a distinct class of organic molecules recognized by long hydrocarbon chains with a terminal carboxyl (–COOH) functional group. The acidic properties and ability to serve as the primary site for chemical interactions are attributed to their carboxyl group [58]. Hence, it enables fatty acids to participate in diverse biochemical reactions essential for their physiological and metabolic functions. In addition, fatty acids provide nutrition and pharmacological functions by serving as energy substrates during periods of limited glucose availability and as precursors for the biosynthesis of hormones and intracellular membrane components, thereby contributing to post-translational protein modification [59].

**Figure 6:**
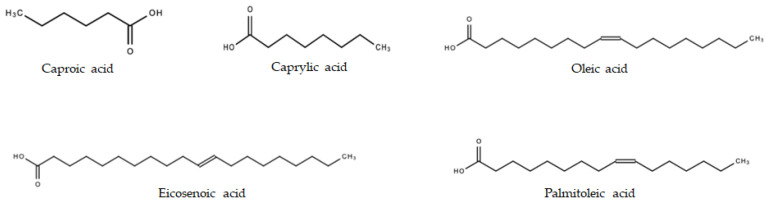
Fatty acids found in *C. sativa*.

The fatty acids of *C. sativa* seed from different countries were compared by Ross and colleagues [60]. Both saturated and unsaturated fatty acids were found in the seed of *C. sativa* across different countries. The fatty acids identified (Figure 6) included caproic, caprylic, myristic, palmitoleic, palmitic, margaric, oleic, linolenic, isolinolenic, linoleic, stearic, eicosenoic, arachidic, isoarachidic, and behenic acids [60].

## 4. Pharmacological Properties of Non-Cannabinoid Phytochemicals

The pharmacological activities of non-cannabinoid phytochemicals are both diverse and therapeutically relevant, contributing significantly to the medicinal potential of various plants beyond the effects attributed solely to cannabinoids. The non-cannabinoid phytochemicals also may act synergistically with other phytoconstituents to enhance therapeutic outcomes or offer distinct mechanisms of action in the management of various pathological conditions, as summarized in Table 1.

### 4.1. Anti-Inflammatory Activity

Inflammation is a fundamental biological process that facilitates the repair of tissue after injury. It involves a sequence of microvascular and cellular responses aimed at eliminating damaged cells and promoting tissue regeneration [61]. This process includes increased permeability of small blood vessels, adhesion of circulating immune cells near the site of injury, migration of various cell types to the affected area, programmed cell death, and the formation of blood vessels and new tissue. However, abnormal inflammatory responses could lead to a group of clinical conditions, such as obesity, atherosclerosis, type 2 diabetes, inflammatory bowel disorders, asthma, neurodegenerative disorders, cancer, and rheumatoid arthritis [62,63].

Previous studies established a positive correlation between the composition of terpenes in *C. sativa* and anti-inflammatory activity. For example, Li and coworkers reported that β-myrcene, a terpene isolated from *C. sativa*, showed significant anti-inflammatory activity by reducing IL6 expression [64]. In another study, the sesquiterpene β-caryophyllene, the most abundant sesquiterpene in *C. sativa*, has been found to bind with cannabinoid receptor type 2 (CB2) and is believed to contribute significantly to the anti-inflammatory activity observed in certain cannabis formulations in mouse models of inflammation [65].

Also, the anti-inflammatory property of flavonoids in *C. sativa* is reported. Cannflavins present in ethanolic extracts of *C. sativa* leaves were earlier observed to suppress prostaglandin E2 (PGE2) production triggered by 12-O-tetradecanoylphorbol-13-acetate (TPA), the proinflammatory agent, in cultured human synovial cells derived from rheumatoid arthritis patients [66]. Recently, cannflavin A and cannflavin B have been reported to demonstrate anti-inflammatory effects by suppressing the activity of 5-lipoxygenase and microsomal prostaglandin E2 synthase-1, which results in decreased leukotrienes and prostaglandin E2 (PGE2) production, respectively [67]. In addition, cannflavin A is reported to elicit minimal inhibition of cyclooxygenase enzymes COX-1 and COX-2; therefore, it decreases the harmful adverse effects commonly associated with traditional COX-inhibiting anti-inflammatory medications, such as gastrointestinal damage [67].

### 4.2. Anti-Cancer Activity

Flavonoids have been widely studied for cancer treatment because they are able to interfere with the growth, survival, proliferation, and migration of cancer cells through various molecular mechanisms. For example, quercetin modulates NF-κB, PKC-δ, ERK1/2, and AMPKα in cancer metastasis [68], while hesperetin reduces transcription and translation of Bcl-2 in PC-3 cell lines [69]. Interestingly, recent research shows that flavonoids from *C. sativa* also exhibit significant anti-cancer activity. FBL-03G, a synthetic isomer of cannflavin B (cannabis flavonoid), effectively delays the progression of both metastatic and local tumors in pancreatic cancer in animal models [70].

Also, apigenin, a flavonoid found in the flowers and leaves of cannabis, is reported to inhibit the proliferation and induce apoptosis by down-regulating BCL-XL through activation of the caspase family in Diffuse large B-cell lymphoma [71]. In preclinical testing, Apigenin suppresses tumor growth by modulating the estrogen receptor (ER)-dependent PI3K/Akt/mTOR signaling pathway in female BALB/c nude mice [72]. Kaempferol, on the other hand, has been reported to reduce tumor growth and metastasis through modulation of epidermal growth factor receptor and glycolysis inhibition by decreasing hexokinase-2 expression in the esophagus squamous cell carcinoma [73]. However, Mamouni and co-workers [74] developed a standardized composition of *C. sativa* flavonoids that comprises luteolin, quercetin, and kaempferol, and investigated their synergistic in vitro cytotoxicity in a prostate cancer model of mice. They discovered that the standardized composition retarded the metastatic spread of prostate cancer cells [74].

The anti-cancer potential of terpenes has been extensively investigated in recent years [75]. Myrcene in *Zanthoxylum rhoifolium* leaves has been reported to have cytotoxic activity against tumoral cells in human colon adenocarcinoma [76]. Recently, myrcene from *C. sativa* has been found to demonstrate anti-tumor activity by decreasing the metabolic activity of the A549 lung adenocarcinoma cells and increasing caspase-3 activity, coupled with a decrease in mitochondrial membrane potential synthesis in the human lung cancer cell line (A549) [77]. Interestingly, an extract that consists of a greater proportion of monoterpenes within the terpene fraction (comprising 19.6% monoterpenes and 80.4% sesquiterpenes) demonstrates markedly greater selectivity for cancer cells over the non-tumorigenic cells, but is less active than cannabinoids that were tested individually [78].

### 4.3. Anti-Diabetes Activity

Diabetes is a metabolic disorder with a multifunctional approach to managing its persistent hyperglycemia and preventing complications. Plants’ phytochemicals have been reported to exhibit an anti-diabetic effect using any or a combination of multifunctional approaches that include improving insulin sensitivity and glucose homeostasis [79], inhibition of carbohydrate-digesting enzymes [80], modulation of key enzymes involved in glucose metabolism [81], protecting pancreatic β-cells and reducing apoptosis [82], and reducing inflammation and oxidative stress [83].

A recent study demonstrated that the ethanolic and aqueous extracts rich in flavonoids from *C. sativa* markedly reduced postprandial blood glucose levels in normal rats and inhibited intestinal α-glucosidase [84]. A similar study suggested that flavonoids present in cannabis exhibit anti-diabetic actions through the reduction in oxidative stress, inflammation, and pancreatic β-cells apoptosis [85]. Studies on anti-diabetic activity on specific flavonoids of *C. sativa* are, however, lacking.

β-caryophyllene, a terpene present in cannabis, has been reported to improve insulin secretion via the activation of small G protein Arf6, Rac1, and Cdc42 in the cannabinoid receptor (type 2) [86]. It also improved glucose uptake and reduced glucose absorption in diabetic rats and RIN-5F cell lines, respectively [87].

### 4.4. Analgesic Activity

Phytochemicals, over the years, have been used extensively for pain management and treatment. The analgesic activity of phytochemicals has been reported to utilize inflammation and modulation [88], reduction in oxidative stress [89], and modulation of pain signaling pathways [90] as their mechanism of action. Non-cannabinoid phytochemicals in *C. sativa*, such as flavonoids, terpenes, and other phenolic compounds, exhibit notable analgesic activity using the above mechanism.

Terpenes found in *C. sativa*, have been reported to demonstrate non-selective interactions with a number of G-protein like coupled receptors (GPCRs) like opioid receptors, cannabinoid receptor type 1 (CB1) and CB2 receptors, GPR55, as well as dopamine, muscarinic, adrenergic, and adenosine receptor that modulate several ion channels, including NMDA, transient receptor potential (TRP) channels, AMPA, kainate, nicotinic, and potassium channels to produce analgesic effects [91]. Specifically, β-myrcene and limonene display analgesic activity by modulation of α2-adrenoreceptors, downregulation of pro-inflammatory cytokines such as TNF-α, blocking of TRPV1 receptors, inhibition of protein kinase A and C, and suppression of the NF-κB/p38 MAPK signaling pathway [92,93].

Limited studies demonstrated the analgesic activity of flavonoids, and a few studies reported that they exert their effects by suppressing both oxidative stress and inflammatory signaling pathways, which contribute immensely to pain. This anti-inflammatory action forms the basis of their potential to regulate neuropathic and inflammatory pain disorders. For example, cannflavin A has been shown to inhibit key inflammatory enzymes like prostaglandin E2, which mediates pain and inflammation [94].

### 4.5. Antimicrobial Activity

Non-cannabinoid compounds in *C. sativa*, predominantly terpenes, are reported to exhibit antibacterial and antifungal activities in vitro [95]. Their proposed mechanisms of action include perturbation of microbial membranes, interference with biofilm formation and quorum-sensing pathways, modulation of efflux pumps, and enzymatic processes. However, minimum inhibitory concentration (MIC) values for purified non-cannabinoid secondary metabolites remain scarce, with most potency data derived from complex plant extracts or essential oil fractions rather than from isolated molecules.

Terpene compounds, including β-caryophyllene, α-pinene, caryophyllene oxide, myrcene, limonene, and β-pinene, have been reported to exhibit antimicrobial activity against clinically relevant microorganisms such as *Acinetobacter calcoaceticus*, *Bacillus subtilis*, *Escherichia coli*, *Staphylococcus aureus*, *Yersinia enterocolitica*, and *Micrococcus luteus* [96,97]. Nissen et al. (2010) reported that the antimicrobial properties of terpenes in *C. sativa* cultivars exhibit significant antimicrobial activity, with particularly strong effects against Gram-positive pathogens belonging to the genera *Enterococcus* and *Streptococcus* [98]. Among the evaluated terpene standards, α-pinene showed the highest antimicrobial efficacy, demonstrating antimicrobial activity against both Gram-negative and Gram-positive bacteria [95].

**Table 1:** The Pharmacological Properties of Non-Cannabinoid Phytochemicals.

Pharmacological Activity	Experimental Model	Class of Phytochemicals	Compound	Outcome	References
Anti-inflammatory	In vitro WI38 lung epithelial cells Animal models of inflammatory (C57BL/6 mice)	Terpene	β-Myrcene	positively correlated (pval = 0.002) with the inhibition of IL6 expression	[64]
	In vitro human peripheral blood assay	Flavonoid	β-Caryophyllene	At a dose of 1 mg kg^−1,^ hyperalgesia gradually reduced thermal sensitivity over the 2-week testing period even up to the basal level	[65]
Anti-cancer	In vitro gastric cancer (GC BGC823 and AGS cells)	Flavonoid	Quercetin-rich extract	Exhibited significant anti-cancer activity against HCT116 cells with an IC_50_ of 60.253 µg/mL.	[68]
	In vitro B-cell lymphoma (U2932 and OCI-LY10 cells) In vivo BALB/c nude mice		FBL-03G (Cannflavin B analog)	Increase in apoptosis and consequential decrease in survival for two pancreatic cancer models- Panc-02 and KPC pancreatic cancer cells treated with varying concentrations of FBL-03G and radiotherapy	[70]
			Apigenin	A significant reduction in the expression of the pro-proliferative pathway PI3K/mTOR	[71,72]
	In vitro human lung cancer cell line (A549) Doxorubicin-induced chronic cardiotoxicity Rats	Terpene	β-Caryophyllene	Activated the JAK1/STAT3 pathway in vitro Decreased doxorubicin-induced cardiotoxicity	[99,100]
	Various cancer cell lines (MV4-11, AGS, HT-29, MDA-MB-468, MCF-7)		Monoterpene/Sesquiterpene mix	IC_50_ (μg/mL) values of 10.04, 7.31, 10.34, and 15.41, respectively	[78]
Anti-diabetic	In vitro α-amylase & α-glucosidase assay Diabetes mice	Flavonoid	Cannabis extracts rich in Flavonoids Ethanol-rich flavonoid extract	Intestinal α-glucosidase activity, with an IC_50_ of 32.23 µg/mL Ameliorate hyperglycemia and improve glucose homeostasis and islet function in STZ-treated mice	[84,85]
		Terpene	β-Caryophyllene		[86,87]
Analgesic	Male Wistar rats and Swiss mice	Terpene	β-Myrcene	Downregulate the KO_2_-induced mRNA expression of gp91phox, cyclooxygenase (COX)-2, and preproendothelin-1. Upregulated KO_2_-reduced nuclear factor (erythroid-derived 2)-like 2 (Nrf2) mRNA expression coupled with enhanced heme oxygenase (HO-1) mRNA expression.	[92]
				Monoterpene	
				Prenylated flavone	
Antimicrobial	Minimal Bactericidal Concentration assay	Flavonoid Terpenes	β-caryophyllene myrcene limonene α-pinene	0.25 mg/mL against *E. coli* 1.35 (% *v*/*v*) against *Pseudomonas savastanoi* 1.39 (% *v*/*v*) against *Enterococcus faecium* 1.67 (% *v*/*v*) against *Clostridium sporogens*	[95,96,98]

## 5. The Entourage Effect of Non-Cannabinoid Phytochemicals of *Cannabis sativa*

The entourage effect describes the synergistic interaction among cannabis phytochemicals, such as cannabinoids and non-cannabinoids, that combine their biological or pharmacological effects to modulate overall therapeutic outcomes compared with the effects of each compound when used in isolation. For instance, Cannabinoids suppress pro-inflammatory cytokines via CB_2_ and PPARγ pathways, while flavonoids and terpenes inhibit COX, LOX, and NF-κB signaling, resulting in additive or synergistic anti-inflammatory effects.

Previous data suggest that Cannabinoids and terpenes converge on transient receptor potential (TRP) channels (TRPV1, TRPA1, TRPM8), serotonin (5-HT_1_A) receptors, GABAergic receptors, and adenosine receptors. This multi-receptor engagement exacerbates synergistic analgesic, anxiolytic, and neuroprotective outcomes [101,102]. Furthermore, CBD and some flavonoids, β-caryophyllene, have been reported to inhibit fatty acid amide hydrolase (FAAH), a membrane-bound enzyme principally responsible for the degradation of anandamide (N-arachidonoylethanolamine), thus increasing endogenous anandamide levels. This amplifies cannabinoid signaling without direct receptor overstimulation [103]. The entourage effect of non-cannabinoid phytochemicals of *C. sativa* is summarized in Table 2.

## 6. Challenges and Limitations

Several preclinical studies emphasize the therapeutic potential of non-cannabinoid phytochemicals derived from *C. sativa*. The most studied non-cannabinoid phytochemicals are flavonoids and terpenes, while studies on alkaloids and phenolic acids are lacking. Promising potentials are reported on the pharmacological activity of the non-cannabinoid phytochemicals, but none of these studies made it to the clinical trials. Preclinical studies specifically investigating isolated non-cannabinoid phytochemicals are limited. The majority of human data comes from trials using whole-plant extracts or formulations that include both cannabinoids and non-cannabinoid constituents, making it difficult to separate and identify the individual effects of the non-cannabinoid compounds.

Also, challenges to clinical translation include regulatory limitations, inconsistent plant chemotypes and extraction methods, and fluctuations in phytochemical composition, which hinder reproducibility, precise dosing, and accurate identification of therapeutic effects. For example, regarding the variability in chemical composition on strain and cultivation methods, a study has demonstrated that the flavonoid content of *C. sativa* strain “Carmagnola Cs” contains up to 25% more compared to varieties such as “Kompolti,” highlighting strain-specific variations [107].

The absence of well-defined reference standards and universally recognized analytical techniques for identifying and quantifying minor non-cannabinoid phytochemicals presents a significant challenge. This limits accurate characterization, purity evaluation, and consistent dosing, in particular, because these compounds are typically present in low concentrations, thus leading to a lack of standardization in phytochemical profiles.

## 7. Materials and Methods

Scopus, Google Scholar, and PubMed were utilized as databases to search for relevant published literature. The search employed the keywords in the following order: *Cannabis sativa* phytochemicals, non-cannabinoid compounds of *Cannabis sativa*, pharmacological activity of non-cannabinoid compounds of *Cannabis sativa*. The total number of published articles initially retrieved between January 2010 and May 2025 was 187. From these, only one subset was selected according to the following inclusion and exclusion criteria.

Inclusion criteria:

1. Peer-reviewed articles that published the pharmacological activity of non-cannabinoid compounds of *C. sativa*.
2. Peer-reviewed articles that published the isolation, extraction, purification, and biological activity of non-cannabinoid compounds of *C. sativa*.
3. Peer-reviewed articles that published the in vitro, preclinical, and clinical studies of non-cannabinoid compounds of *C. sativa*.
4. The exclusion criteria were set to exclude the following articles.
5. Peer-reviewed articles that published the cannabinoids phytochemicals of *C. sativa* with respect to their extraction, isolation, biological, and pharmacological activity.
6. Peer-reviewed articles that published the non-cannabinoids that are not extracted from *C. sativa*.

**Figure 7:**
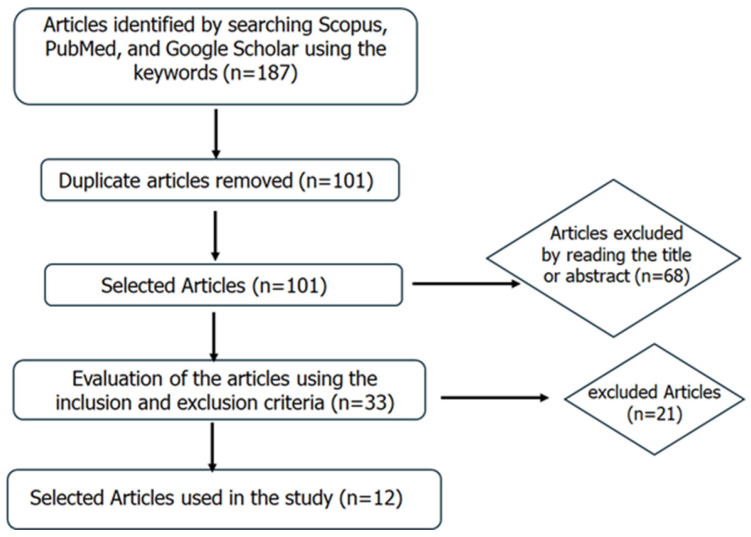
Search results of the articles selected for review.

Following the inclusion and exclusion criteria, 12 articles were selected for the study. The findings of the search are presented in Figure 7 below.

## 8. Conclusions and Perspectives

The growing body of evidence on the non-cannabinoid bioactivity highlights their roles as integral contributors to the therapeutic profile of *C. sativa*. However, the absence of accurate characterization, purity evaluation, lack of clinical trials, and standardization masks the therapeutic potential. Their diverse mechanisms of action, coupled with their potential to modulate cannabinoid effects, make them potential candidates for future drug development and, in particular, the formulation of cannabinoid-based therapeutics. Further research into non-cannabinoid interactions, pharmacokinetics, and clinical efficacy is warranted to optimize the medical application of cannabis. Such synergy may underlie enhanced therapeutic outcomes and reduced adverse effects observed with full-spectrum cannabis extracts compared to isolated cannabinoids.

## Figures and Tables

**Table 2:** The entourage effect of non-cannabinoid phytochemicals of *Cannabis sativa*.

Targets	Non-Cannabinoid Entourage Effect	Interaction in the Entourage Effect	References
Terpenes: myrcene, limonene, β-caryophyllene; Flavonoids: cannflavins A & B	Terpenes: myrcene, limonene, β-caryophyllene; Flavonoids: cannflavins A & B	Specific pairings (e.g., CBD + myrcene) can alter pharmacodynamics and subjective effects.	[101,102]
β-Caryophyllene and Terpinolene	Olfactory receptors, serotonin receptors, GABAergic systems, enzymes like COX	Influence receptor binding affinity, signal transduction, and downstream effects.	[104]
CBD and β-caryophyllene	inhibit fatty acid amide hydrolase (FAAH)	Modifiers of cannabinoid signaling, often enhancing or balancing therapeutic profiles.	[103]
THC + CBD + β-caryophyllene	Terpenes and flavonoids show efficacy in animal models (e.g., anti-inflammatory effects).	reduced anxiety, improved pain	[105]
Terpenes/terpenoids	Terpenes can inhibit/induce CYP450	Co-presence influences pharmacokinetics such as absorption, distribution, metabolism, and elimination.	[106]

## Data Availability

No new data were created or analyzed in this study. Data sharing is not applicable to this article.

## References

[1] Chaachouay N., Qureshi R., Benkhnigue O., Azeroual A., Zidane L. (2024). Marijuana (*Cannabis sativa* L. Cannabaceae). Comprehensive Guide to Hallucinogenic Plants.

[2] Maule W. (2015). Medical uses of marijuana (*Cannabis sativa*): Fact or fallacy?. Br. J. Biomed. Sci..

[3] Omare M.O., Kibet J.K., Cherutoi J.K., Kengara F.O. (2021). Current trends in the use of *Cannabis sativa*: Beyond recreational and medicinal applications. Open Access Libr. J..

[4] Baral P., Bagul V., Gajbhiye S. (2020). Hemp seed oil for skin care (non-drug *Cannabis sativa* L.): A review. World J. Pharm. Res..

[5] Arango S., Greco R., Guzzo N., Raffrenato E., Montanari M., Bailoni L. (2023). Physical characterization of ten hemp varieties to use as animal bedding material. Animals.

[6] Pino S., Espinoza L., Jara-Gutiérrez C., Villena J., Olea A.F., Díaz K. (2023). Study of cannabis oils obtained from three varieties of *C. sativa* and by two different extraction methods: Phytochemical characterization and biological activities. Plants.

[7] Radwan M.M., Chandra S., Gul S., ElSohly M.A. (2021). Cannabinoids, phenolics, terpenes and alkaloids of cannabis. Molecules.

[8] Cásedas G., Moliner C., Maggi F., Mazzara E., López V. (2022). Evaluation of two different *Cannabis sativa* L. extracts as antioxidant and neuroprotective agents. Front. Pharmacol..

[9] Naz S., Kashif A.R., Tawab A., Rasool M.Z., Rauf A., Hussain S., Khan U. (2023). Assessment of the antidiabetic properties of essential oil from *Cannabis sativa*. J. Adv. Nutr. Sci. Technol..

[10] Crocq M.-A. (2020). History of cannabis and the endocannabinoid system. Dialogues Clin. Neurosci..

[11] Pisanti S., Bifulco M. (2019). Medical Cannabis: A plurimillennial history of an evergreen. J. Cell. Physiol..

[12] Lata S., Sharma S., Maurya C. (2022). Traditional uses of plants in various rituals and ceremonies among tharu tribe of Udham Singh Nagar, Kumaun Himalaya Uttarakhand, India. Plant Arch..

[13] Bennett C. (2014). The magic and ceremonial use of cannabis in the ancient world. Seeking the Sacred with Psychoactive Substances.

[14] Schultes R.E. (1969). Hallucinogens of plant origin: Interdisciplinary studies of plants sacred in primitive cultures yield results of academic and practical interest. Science.

[15] Peeri H., Koltai H. (2023). Phytocannabinoids have cytotoxic, antiproliferative, and antimigratory activities on cancer cells and cancer stem cells. Cannabis Use, Neurobiology, Psychology, and Treatment.

[16] Wahid M., Ali A., Saqib F., Aleem A., Bibi S., Afzal K., Ali A., Baig A., Khan S.A., Bin Asad M.H.H. (2020). Pharmacological exploration of traditional plants for the treatment of neurodegenerative disorders. Phytother. Res..

[17] Avoseh F.T., Mtunzi F.M., Avoseh O.N., Takaidza S. (2025). *Cannabis sativa*; Ethnobotanicals, Classifications, Pharmacology, and Phytochemistry. Nat. Prod. Commun..

[18] Hussain A., Abidi S.H., Syed Q., Saeed A. (2022). Current knowledge on ethnobotany, phytochemistry and biological activities of Cannabis (hemp) from Pakistan with emphasis on its legalization and regulation: Current knowledge on Cannabis from Pakistan. Ethnobot. Res. Appl..

[19] Kotecka-Majchrzak K., Sumara A., Fornal E., Montowska M. (2020). Oilseed proteins–Properties and application as a food ingredient. Trends Food Sci. Technol..

[20] Mattila P., Mäkinen S., Eurola M., Jalava T., Pihlava J.-M., Hellström J., Pihlanto A. (2018). Nutritional value of commercial protein-rich plant products. Plant Foods Hum. Nutr..

[21] Lu X., Clarke R.C. (1995). The cultivation and use of hemp (*Cannabis sativa* L.) in ancient China. J. Int. Hemp Assoc..

[22] Sharma P.K., Chauhan N., Lal B., Husaini A., Teixeira da Silva J. (2010). Conservation of phyto-diversity of Parvati Valley in northwestern Himalayas of Himachal Pradesh-India. Med. Aromat. Plant Sci. Biotechnol..

[23] Bhatla S.C., Lal M.A. (2023). Secondary metabolites. Plant Physiology, Development and Metabolism.

[24] Khan A., Kanwal F., Ullah S., Fahad M., Tariq L., Altaf M.T., Riaz A., Zhang G. (2025). Plant secondary metabolites—Central regulators against abiotic and biotic stresses. Metabolites.

[25] Montesinos-Navarro A., Pérez-Clemente R.M., Sánchez-Martín R., Gomez-Cadenas A., Verdú M. (2020). Phylogenetic analysis of secondary metabolites in a plant community provides evidence for trade-offs between biotic and abiotic stress tolerance. Evol. Ecol..

[26] Al-Khayri J.M., Rashmi R., Toppo V., Chole P.B., Banadka A., Sudheer W.N., Nagella P., Shehata W.F., Al-Mssallem M.Q., Alessa F.M. (2023). Plant secondary metabolites: The weapons for biotic stress management. Metabolites.

[27] Del Rosario-Makridis G.N. (2023). Molecular Regulation of Glandular Trichome Initiation and Morphogenesis in *Cannabis sativa* L.. Ph.D. Thesis.

[28] Wiley J.L., Lefever T.W., Marusich J.A., Grabenauer M., Moore K.N., Huffman J.W., Thomas B.F. (2016). Evaluation of first generation synthetic cannabinoids on binding at non-cannabinoid receptors and in a battery of in vivo assays in mice. Neuropharmacology.

[29] Stasiłowicz A., Tomala A., Podolak I., Cielecka-Piontek J. (2021). *Cannabis sativa* L. as a natural drug meeting the criteria of a multitarget approach to treatment. Int. J. Mol. Sci..

[30] Stasiłowicz-Krzemień A., Nogalska W., Maszewska Z., Maleszka M., Dobroń M., Szary A., Kępa A., Żarowski M., Hojan K., Lukowicz M. (2024). The use of compounds derived from *Cannabis sativa* in the treatment of epilepsy, painful conditions, and neuropsychiatric and neurodegenerative disorders. Int. J. Mol. Sci..

[31] Keating G.M.J.D. (2017). Delta-9-tetrahydrocannabinol/cannabidiol oromucosal spray (Sativex^®^): A review in multiple sclerosis-related spasticity. Drugs.

[32] Breijyeh Z., Jubeh B., Bufo S.A., Karaman R., Scrano L.J.T. (2021). Cannabis: A toxin-producing plant with potential therapeutic uses. Toxins.

[33] Al-Khazaleh A.K., Zhou X., Bhuyan D.J., Münch G.W., Al-Dalabeeh E.A., Jaye K., Chang D. (2024). The neurotherapeutic arsenal in *Cannabis sativa*: Insights into anti-neuroinflammatory and neuroprotective activity and potential entourage effects. Molecules.

[34] Lowe H., Toyang N., Steele B., Bryant J., Ngwa W. (2021). The endocannabinoid system: A potential target for the treatment of various diseases. Int. J. Mol. Sci..

[35] Pollastro F., Minassi A., Fresu L.G. (2018). Cannabis phenolics and their bioactivities. Curr. Med. Chem..

[36] Dias M.C., Pinto D.C., Silva A.M. (2021). Plant flavonoids: Chemical characteristics and biological activity. Molecules.

[37] Boucher R., Germain H., Desgagné-Penix I. (2025). Exploring the Lesser-Known Bioactive Natural Products of Plant Species of the Genus Cannabis L.: Alkaloids, Phenolic Compounds, and Their Therapeutic Potential. Plants.

[38] Abdel-Kader M.S., Radwan M.M., Metwaly A.M., Eissa I.H., Hazekamp A., ElSohly M.A., Research C. (2023). Chemistry and biological activities of cannflavins of the cannabis plant. Cannabis Cannabinoid Res..

[39] Lowe H., Steele B., Bryant J., Toyang N., Ngwa W. (2021). Non-cannabinoid metabolites of *Cannabis sativa* L. with therapeutic potential. Plants.

[40] Singh R., Singh B., Singh A., Rana S., Sharma K., Viswakarma P., Gopu B., Nalli Y. (2024). Canniprene B, a new prenylated dihydrostilbene with cytotoxic activities from the leaves of *Cannabis sativa*. Nat. Prod. Res..

[41] O’Connor S.E. (2010). 1.25—Alkaloids. Comprehensive Natural Products II.

[42] Divekar P.A., Narayana S., Divekar B.A., Kumar R., Gadratagi B.G., Ray A., Singh A.K., Rani V., Singh V., Singh A.K. (2022). Plant secondary metabolites as defense tools against herbivores for sustainable crop protection. Int. J. Mol. Sci..

[43] Mechoulam R. (1989). Alkaloids in *Cannabis sativa* L.. The Alkaloids: Chemistry and Pharmacology.

[44] Saleem A., Akhtar M.F., Sharif A., Akhtar B., Siddique R., Ashraf G.M., Alghamdi B.S., Alharthy S.A. (2022). Anticancer, cardio-protective and anti-inflammatory potential of natural-sources-derived phenolic acids. Molecules.

[45] Van Nies J.A., De Jong Z., van der Helm-van Mil A.H., Knevel R., Le Cessie S., Huizinga T.W. (2010). Improved treatment strategies reduce the increased mortality risk in early RA patients. Rheumatology.

[46] Rashmi H.B., Negi P.S. (2020). Phenolic acids from vegetables: A review on processing stability and health benefits. Food Res. Int..

[47] Oh C.M., Choi J.Y., Bae I.A., Kim H.T., Hong S.S., Noah J.K., Boo Y.C. (2022). Identification of p-coumaric acid and ethyl p-coumarate as the main phenolic components of hemp (*Cannabis sativa* L.) roots. Molecules.

[48] Kalinowska M., Płońska A., Trusiak M., Gołębiewska E., Gorlewska-Pietluszenko A. (2022). Comparing the extraction methods, chemical composition, phenolic contents and antioxidant activity of edible oils from *Cannabis sativa* and *Silybum marianu* seeds. Sci. Rep..

[49] Benkirane C., Mansouri F., Ben Moumen A., Taaifi Y., Melhaoui R., Caid H.S., Fauconnier M.-L., Elamrani A., Abid M. (2023). Phenolic profiles of non-industrial hemp (*Cannabis sativa* L.) seed varieties collected from four different Moroccan regions. Int. J. Food Sci. Technol..

[50] Nalli Y., Bharti S., Amin T., Singh R., Behera J., Bhayye S.S., Bharitkar Y.P., Goswami A., Verma M.K. (2024). Bioassay-guided fractionations of *Cannabis sativa* extract and HPLC-assisted purifications of anti-proliferative active fractions lead to the isolation of 16 known and one new phytomolecule and their in-silico analysis. Med. Chem. Res..

[51] Turner C.E., Elsohly M.A., Boeren E.G. (1980). Constituents of *Cannabis sativa* L. XVII. A review of the natural constituents. J. Nat. Prod..

[52] Chen B., Cai G., Yuan Y., Li T., He Q., He J. (2012). Chemical constituents in hemp pectin I. Zhongguo Shiyan Fangjixue Zazhi.

[53] Hammond C.T., Mahlberg P.G. (1994). Phloroglucinol glucoside as a natural constituent of *Cannabis sativa*. Phytochemistry.

[54] Chacon F.T., Raup-Konsavage W.M., Vrana K.E., Kellogg J.J. (2022). Secondary terpenes in *Cannabis sativa* L.: Synthesis and synergy. Biomedicines.

[55] Pertwee R.G. (2014). Handbook of Cannabis.

[56] Ben-Shabat S., Fride E., Sheskin T., Tamiri T., Rhee M.-H., Vogel Z., Bisogno T., De Petrocellis L., Di Marzo V., Mechoulam R. (1998). An entourage effect: Inactive endogenous fatty acid glycerol esters enhance 2-arachidonoyl-glycerol cannabinoid activity. Eur. J. Pharmacol..

[57] Russo E.B. (2011). Taming THC: Potential cannabis synergy and phytocannabinoid-terpenoid entourage effects. Br. J. Pharmacol..

[58] Zhu S., He Y., Lei J.-N., Liu Y.-F., Xu Y.-J. (2025). The chemical and biological characteristics of fatty acid esters of hydroxyl fatty acids. Nutr. Rev..

[59] Yang Y.-H., Wen R., Yang N., Zhang T.-N., Liu C.-F. (2023). Roles of protein post-translational modifications in glucose and lipid metabolism: Mechanisms and perspectives. Mol. Med..

[60] Ross S.A., ElSohly H.N., ElKashoury E.A., ElSohly M.A. (1996). Fatty acids of cannabis seeds. Phytochem. Anal..

[61] Eming S.A., Wynn T.A., Martin P. (2017). Inflammation and metabolism in tissue repair and regeneration. Science.

[62] Rajendran P., Chen Y.F., Chen Y.F., Chung L.C., Tamilselvi S., Shen C.Y., Day C.H., Chen R.J., Viswanadha V.P., Kuo W.W. (2018). The multifaceted link between inflammation and human diseases. J. Cell. Physiol..

[63] Bennett J.M., Reeves G., Billman G.E., Sturmberg J.P. (2018). Inflammation–nature’s way to efficiently respond to all types of challenges: Implications for understanding and managing “the epidemic” of chronic diseases. Front. Med..

[64] Li D., Ilnytskyy Y., Ghasemi Gojani E., Kovalchuk O., Kovalchuk I. (2022). Analysis of anti-cancer and anti-inflammatory properties of 25 high-THC cannabis extracts. Molecules.

[65] Klauke A.-L., Racz I., Pradier B., Markert A., Zimmer A., Gertsch J., Zimmer A. (2014). The cannabinoid CB2 receptor-selective phytocannabinoid beta-caryophyllene exerts analgesic effects in mouse models of inflammatory and neuropathic pain. Eur. Neuropsychopharmacol..

[66] Barrett M., Gordon D., Evans F. (1985). Isolation from *Cannabis sativa* L. of cannflavin—A novel inhibitor of prostaglandin production. Biochem. Pharmacol..

[67] Werz O., Seegers J., Schaible A.M., Weinigel C., Barz D., Koeberle A., Allegrone G., Pollastro F., Zampieri L., Grassi G. (2014). Cannflavins from hemp sprouts, a novel cannabinoid-free hemp food product, target microsomal prostaglandin E2 synthase-1 and 5-lipoxygenase. PharmaNutrition.

[68] Li H., Chen C. (2018). Quercetin has antimetastatic effects on gastric cancer cells via the interruption of uPA/uPAR function by modulating NF-κb, PKC-δ, ERK1/2, and AMPKα. Integr. Cancer Ther..

[69] Sambantham S., Radha M., Paramasivam A., Anandan B., Malathi R., Chandra S.R., Jayaraman G. (2013). Molecular mechanism underlying hesperetin-induced apoptosis by in silico analysis and in prostate cancer PC-3 cells. Asian Pac. J. Cancer Prev..

[70] Moreau M., Ibeh U., Decosmo K., Bih N., Yasmin-Karim S., Toyang N., Lowe H., Ngwa W. (2019). Flavonoid derivative of cannabis demonstrates therapeutic potential in preclinical models of metastatic pancreatic cancer. Front. Oncol..

[71] Huang S., Yu M., Shi N., Zhou Y., Li F., Li X., Huang X., Jin J. (2020). Apigenin and Abivertinib, a novel BTK inhibitor synergize to inhibit diffuse large B-cell lymphoma in vivo and vitro. J. Cancer.

[72] Zhang E., Zhang Y., Fan Z., Cheng L., Han S., Che H. (2020). Apigenin inhibits histamine-induced cervical cancer tumor growth by regulating estrogen receptor expression. Molecules.

[73] Yao S., Wang X., Li C., Zhao T., Jin H., Fang W. (2016). Kaempferol inhibits cell proliferation and glycolysis in esophagus squamous cell carcinoma via targeting EGFR signaling pathway. Tumor Biol..

[74] Mamouni K., Zhang S., Li X., Chen Y., Yang Y., Kim J., Bartlett M.G., Coleman I.M., Nelson P.S., Kucuk O. (2018). A novel flavonoid composition targets androgen receptor signaling and inhibits prostate cancer growth in preclinical models. Neoplasia.

[75] Kamran S., Sinniah A., Abdulghani M.A., Alshawsh M.A. (2022). Therapeutic potential of certain terpenoids as anticancer agents: A scoping review. Cancers.

[76] Silva S.L.d., Figueiredo P.M., Yano T. (2007). Cytotoxic evaluation of essential oil from *Zanthoxylum rhoifolium* Lam. leaves. Acta Amaz..

[77] Bai X., Tang J. (2020). Myrcene exhibits antitumor activity against lung cancer cells by inducing oxidative stress and apoptosis mechanisms. Nat. Prod. Commun..

[78] Haczkiewicz M., Świtalska M., Łyczko J., Pluta M., Wietrzyk J., Gliszczyńska A. (2025). Extraction of Cannabinoids and Terpenes from Hemp Flowers and Leaves (*Cannabis sativa* L., Futura 75): Chemical Profiling and Evaluation of Anticancer Properties. Molecules.

[79] Li M., Chi X., Wang Y., Setrerrahmane S., Xie W., Xu H. (2022). Trends in insulin resistance: Insights into mechanisms and therapeutic strategy. Signal Transduct. Ther..

[80] Bhujle R.R., Nayak N., Gowda N.N., Pandiselvam R., Sunil C.K. (2025). A comprehensive review on influence of millet processing on carbohydrate-digesting enzyme inhibitors and implications for diabetes management. Crit. Rev. Biotechnol..

[81] Zhang Y., Li Q., Huang Z., Li B., Nice E.C., Huang C., Wei L., Zou B. (2022). Targeting glucose metabolism enzymes in cancer treatment: Current and emerging strategies. Cancers.

[82] Prasad M.K., Mohandas S., Ramkumar K.M. (2023). Dysfunctions, molecular mechanisms, and therapeutic strategies of pancreatic β-cells in diabetes. Apoptosis.

[83] Teodoro J.S., Nunes S., Rolo A.P., Reis F., Palmeira C.M. (2019). Therapeutic options targeting oxidative stress, mitochondrial dysfunction and inflammation to hinder the progression of vascular complications of diabetes. Front. Physiol..

[84] Haddou S., Elrherabi A., Loukili E.H., Abdnim R., Hbika A., Bouhrim M., Al Kamaly O., Saleh A., Shahat A.A., Bnouham M. (2023). Chemical analysis of the antihyperglycemic, and pancreatic α-amylase, lipase, and intestinal α-glucosidase inhibitory activities of *Cannabis sativa* L. seed extracts. Molecules.

[85] Kim Y., Kim W., Kim S.-H., Sim K.-S., Kim K.-H., Cho K.-H., Kwon G.-S., Lee J.-B., Kim J.-H. (2023). Protective effects of hemp (*Cannabis sativa*) root extracts against insulin-deficient diabetes mellitus in mice. Molecules.

[86] Suijun W., Zhen Y., Ying G., Yanfang W. (2014). A role for trans-caryophyllene in the moderation of insulin secretion. Biochem. Biophys. Res. Commun..

[87] Kumawat V.S., Kaur G. (2020). Insulinotropic and antidiabetic effects of β-caryophyllene with l-arginine in type 2 diabetic rats. J. Food Biochem..

[88] Silva-Correa C.R., Campos-Reyna J.L., Villarreal-La Torre V.E., Calderón-Peña A.A., Blas M.V.G., Aspajo-Villalaz C.L., Cruzado-Razco J.L., Sagástegui-Guarniz W.A., Guerrero-Espino L.M. (2021). Potential activity of medicinal plants as pain modulators: A review. Pharmacogn. J..

[89] Manchope M.F., Calixto-Campos C., Coelho-Silva L., Zarpelon A.C., Pinho-Ribeiro F.A., Georgetti S.R., Baracat M.M., Casagrande R., Verri W.A. (2016). Naringenin inhibits superoxide anion-induced inflammatory pain: Role of oxidative stress, cytokines, Nrf-2 and the NO−cGMP−PKG−KATPChannel signaling pathway. PLoS ONE.

[90] Mota C.M., Rodrigues-Santos C., Carolino R.O., Anselmo-Franci J.A., Branco L.G. (2020). Citral-induced analgesia is associated with increased spinal serotonin, reduced spinal nociceptive signaling, and reduced systemic oxidative stress in arthritis. J. Ethnopharmacol..

[91] Russo E.B. (2019). The case for the entourage effect and conventional breeding of clinical cannabis: No “strain,” no gain. Front. Plant Sci..

[92] Pereira E.W., Heimfarth L., Santos T.K., Passos F.R., Siqueira-Lima P., Scotti L., Scotti M.T., da Silva Almeida J.R.G., Campos A.R., Coutinho H.D. (2022). Limonene, a citrus monoterpene, non-complexed and complexed with hydroxypropyl-β-cyclodextrin attenuates acute and chronic orofacial nociception in rodents: Evidence for involvement of the PKA and PKC pathway. Phytomedicine.

[93] Lorenzetti B.B., Souza G.E., Sarti S.J., Santos Filho D., Ferreira S.H. (1991). Myrcene mimics the peripheral analgesic activity of lemongrass tea. J. Ethnopharmacol..

[94] Elikottil J., Gupta P., Gupta K. (2009). The analgesic potential of cannabinoids. J. Opioid Manag..

[95] Guimarães A.C., Meireles L.M., Lemos M.F., Guimarães M.C.C., Endringer D.C., Fronza M., Scherer R. (2019). Antibacterial activity of terpenes and terpenoids present in essential oils. Molecules.

[96] Schofs L., Sparo M.D., Sanchez Bruni S.F. (2021). The antimicrobial effect behind *Cannabis sativa*. Pharmacol. Res. Perspect..

[97] da Silva Rivas A.C., Lopes P.M., de Azevedo Barros M.M., Costa Machado D.C., Alviano C.S., Alviano D.S. (2012). Biological activities of α-pinene and β-pinene enantiomers. Molecules.

[98] Nissen L., Zatta A., Stefanini I., Grandi S., Sgorbati B., Biavati B., Monti A. (2010). Characterization and antimicrobial activity of essential oils of industrial hemp varieties (*Cannabis sativa* L.). Fitoterapia.

[99] Meeran M.N., Al Taee H., Azimullah S., Tariq S., Adeghate E., Ojha S. (2019). β-Caryophyllene, a natural bicyclic sesquiterpene attenuates doxorubicin-induced chronic cardiotoxicity via activation of myocardial cannabinoid type-2 (CB2) receptors in rats. Chem.-Biol. Interact..

[100] Hanušová V., Caltová K., Svobodová H., Ambrož M., Skarka A., Murínová N., Králová V., Tomšík P., Skálová L. (2017). The effects of β-caryophyllene oxide and trans-nerolidol on the efficacy of doxorubicin in breast cancer cells and breast tumor-bearing mice. Biomed. Pharmacother..

[101] Janero D.R., Makriyannis A. (2014). Terpenes and lipids of the endocannabinoid and transient-receptor-potential-channel biosignaling systems. ACS Chem. Neurosci..

[102] Heblinski M., Santiago M., Fletcher C., Stuart J., Connor M., McGregor I.S., Arnold J.C. (2020). Terpenoids commonly found in *Cannabis sativa* do not modulate the actions of phytocannabinoids or endocannabinoids on TRPA1 and TRPV1 channels. Cannabis Cannabinoid Res..

[103] Criscuolo E., De Sciscio M.L., Fezza F., Maccarrone M. (2020). In silico and in vitro analysis of major cannabis-derived compounds as fatty acid amide hydrolase inhibitors. Molecules.

[104] Zou S., Kumar U. (2018). Cannabinoid receptors and the endocannabinoid system: Signaling and function in the central nervous system. Int. J. Mol. Sci..

[105] Downer E. (2020). Anti-inflammatory potential of terpenes present in *Cannabis sativa* L.. ACS Chem. Neurosci..

[106] Auxtero M.D., Chalante S., Abade M.R., Jorge R., Fernandes A. (2021). Potential herb–drug interactions in the management of age-related cognitive dysfunction. Pharmaceutics.

[107] Brousseau V.D., Wu B.-S., MacPherson S., Morello V., Lefsrud M. (2021). Cannabinoids and terpenes: How production of photo-protectants can be manipulated to enhance *Cannabis sativa* L. phytochemistry. Front. Plant Sci..

